# *In Vitro* Screening of Environmental Chemicals for Targeted Testing Prioritization: The ToxCast Project

**DOI:** 10.1289/ehp.0901392

**Published:** 2009-12-14

**Authors:** Richard S. Judson, Keith A. Houck, Robert J. Kavlock, Thomas B. Knudsen, Matthew T. Martin, Holly M. Mortensen, David M. Reif, Daniel M. Rotroff, Imran Shah, Ann M. Richard, David J. Dix

**Affiliations:** National Center for Computational Toxicology, Office of Research and Development, U.S. Environmental Protection Agency, Research Triangle Park, North Carolina, USA

**Keywords:** in vitro screening, liver proliferative lesions, liver tumors, pathways, ToxCast

## Abstract

**Background:**

Chemical toxicity testing is being transformed by advances in biology and computer modeling, concerns over animal use, and the thousands of environmental chemicals lacking toxicity data. The U.S. Environmental Protection Agency’s ToxCast program aims to address these concerns by screening and prioritizing chemicals for potential human toxicity using *in vitro* assays and *in silico* approaches.

**Objectives:**

This project aims to evaluate the use of *in vitro* assays for understanding the types of molecular and pathway perturbations caused by environmental chemicals and to build initial prioritization models of *in vivo* toxicity.

**Methods:**

We tested 309 mostly pesticide active chemicals in 467 assays across nine technologies, including high-throughput cell-free assays and cell-based assays, in multiple human primary cells and cell lines plus rat primary hepatocytes. Both individual and composite scores for effects on genes and pathways were analyzed.

**Results:**

Chemicals displayed a broad spectrum of activity at the molecular and pathway levels. We saw many expected interactions, including endocrine and xenobiotic metabolism enzyme activity. Chemicals ranged in promiscuity across pathways, from no activity to affecting dozens of pathways. We found a statistically significant inverse association between the number of pathways perturbed by a chemical at low *in vitro* concentrations and the lowest *in vivo* dose at which a chemical causes toxicity. We also found associations between a small set of *in vitro* assays and rodent liver lesion formation.

**Conclusions:**

This approach promises to provide meaningful data on the thousands of untested environmental chemicals and to guide targeted testing of environmental contaminants.

There are thousands of environmental chemicals, including many industrial chemicals and pesticidal active and inert ingredients, with the potential for significant human exposures but for which toxicity information is either limited or nonexistent (Judson et al. 2009). This data gap is due largely to the high cost and length of time required to conduct animal testing in rodents and other species. A complete set of regulatory tests for a single chemical (including those for carcinogenicity and for chronic, reproductive, and development toxicity) uses thousands of animals and costs millions of dollars. In addition, traditional animal tests often yield limited information on mechanism of action, and hence on the cellular pathways that could lead to toxicity in humans. Such mechanistic information is key to moving beyond default approaches for extrapolating from high-dose animal toxicity tests to estimation of human risk at realistic exposure levels.

There is a pressing need to screen the large backlog of chemicals for their potential toxicity and, ultimately, their contribution to human diseases. The National Research Council (2007) advocated the use of mechanistically informative *in vitro* assays based on human cells or human cell constituents that measure effects on “toxicity pathways” leading to human disease. The U.S. Environmental Protection Agency (EPA), through its ToxCast program (Dix et al. 2007) and the Tox21 collaboration with the National Toxicology Program and the National Institutes of Health Chemical Genomics Center, is pursuing similar objectives and applying many of the ideas represented in the National Research Council report (Collins et al. 2008; Kavlock et al. 2009).

ToxCast is a large-scale experiment using a battery of *in vitro*, high-throughput screening (HTS) assays, applied to a relatively large and diverse chemical space, to develop methods to predict potential toxicity of environmental chemicals at a fraction of the cost of full-scale animal testing. Three major goals of ToxCast are to *a*) identify *in vitro* assays that can reliably indicate alterations in biological processes of relevance to *in vivo* toxicity; *b*) develop signatures or prediction models based on multiple assays, along with computed or available chemical properties, that can achieve higher predictive power than single assays or chemical structure alone; and *c*) use these combined *in silico* and *in vitro* assay-based signatures to screen large numbers of previously untested environmental chemicals. The ToxCast data set provides a rich resource for identifying chemically induced changes in biological pathways that are associated with *in vivo* end points and that could potentially lead to human disease. Chemicals whose properties and assay profiles match these predictive signatures can be prioritized for more in-depth testing, which may include nontraditional, mechanism-focused *in vivo* tests. In this article, we provide an overview of the entire ToxCast phase I assay results data set and present initial analyses and findings.

## Materials and Methods

Phase I of ToxCast employed a chemical library of 320 substances (U.S. EPA 2008a). Within this set there are 309 unique chemicals, most of which are food-use pesticides for which extensive animal testing results are available. The mechanisms of toxicity for a number of these chemicals are known, thus affording the opportunity to match *in vitro* results with existing knowledge. Further information on the chemical library is provided in the Supplemental Material (available online at doi:10.1289/ehp.0901392).

We screened the chemical library using nine separate assay technologies, with assays run in concentration–response format and in some cases with multiple time points. Assays encompass both direct, primary interactions between chemicals and molecular targets and downstream cellular events such as gene expression. Table 1 summarizes the nine *in vitro* assay technologies, and Supplemental Material, Table 1 (doi:10.1289/ehp.0901392) lists the complete set of *in vitro* assays. There are 467 cell-free or cell-based assays. Assay sets include biochemical HTS and cell-based HTS assays measuring direct molecular interactions with specific protein targets; high-content cell-imaging assays measuring complex cellular phenotypes; a multiplexed gene expression assay for xenobiotic metabolizing enzymes and transporters in human primary hepatocytes; multiplexed transcription factor reporter assays; multiplexed biological activity profiling assays measuring biomarkers in a variety of human primary cell cocultures; assays measuring effects of phase I and II xenobiotic metabolizing enzyme (XMEs) on cytotoxicity; an HTS genotoxicity assay; and a real-time cellular impedance assay that measures the kinetics of cell growth and changes in morphology. For all cell-based assays, time points were selected on the basis of studies conducted during the assay development and were considered optimal for the end point being evaluated.

A total of 624 *in vitro* assay end points (including multiple time points) were measured for each chemical, generating > 200,000 concentration responses. Assays have been mapped to a total of 315 genes, most of which are human (231) or rat (65) [see Supplemental Material, Table 1 (doi:10.1289/ehp.0901392)]. In all cases we report a characteristic micromolar concentration for each chemical–assay combination. These values were either half-maximal activity concentration (AC_50_) or lowest effective concentration (LEC) at which there was a statistically significant change from the concurrent negative control. Criteria for determining the characteristic concentration is given in the Supplemental Material. Chemical–assay combinations that did not show significant activity below the highest concentration tested were labeled inactive. The complete data set, including AC_50_/LEC values for all chemical–assay measurement pairs, is available from the EPA ToxCast Web site (U.S. EPA 2008b). Experimental protocols and information on data quality are summarized in the Supplemental Material.

Many of the compounds in our library have matching guideline study animal toxicity data. Information from regulatory toxicity studies on the pesticide compounds submitted to the U.S. EPA (Knudsen et al. 2009; Martin et al. 2009a, 2009b) were compiled in the U.S. EPA Toxicity Reference Database (ToxRefDB) (U.S. EPA 2008c). Study types include rat and mouse 2-year cancer or chronic bioassays, rat multigenerational reproductive toxicity assays, and rat and rabbit prenatal developmental toxicity assays. ToxRefDB provides the lowest effective level at which particular *in vivo* treatment-related effects were significantly different from negative controls. For each of the *in vivo* study types, typically 250–280 of the ToxCast chemicals had data available and entered into ToxRefDB.

## Results

**Figure 1:**
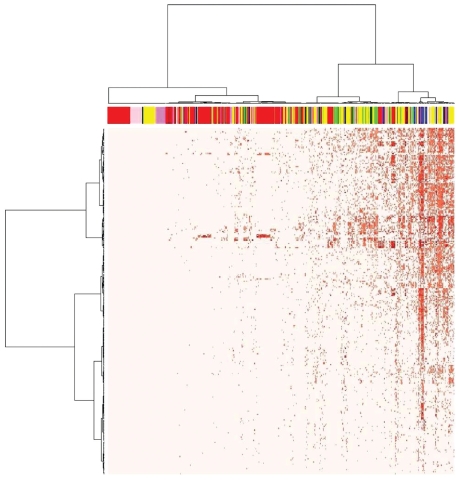
Heat map of 624 assay measurements (including multiple time points where available) in ToxCast phase I data set. Assays are arranged left to right, and chemicals are arranged top to bottom. The color bar at the top indicates the assay type: red (cell-free HTS), violet (multiplexed transcription reporter), yellow (biologically multiplexed activity profiling), green (high-content cell imaging), blue (multiplexed gene expression), pink (cell-based HTS), black (phase I and II XME cytotoxicity), white (real-time cell electronic sensing), and orange (HTS genotoxicity). Data values are –log_10_(AC_50_/LEC), where light pink is inactive and darker reds indicate increased activity (lower AC_50_/LEC).

Figure 1 shows a heat map of the entire *in vitro* data set, providing an overview of the data. Generally, the biochemical HTS assays (indicated by red in the top band) had fewer hits than did the cell-based assays, as evident from the increasing density of hits progressing from left to right in the heat map. On the left side of this plot are 87 assays that had no AC_50_/LEC values identified for any of the chemicals at levels below the highest concentration tested (see Table 1 for concentration ranges tested). In Figure 1, all hits are shown, up to where the AC_50_/LEC occurred at the highest tested concentration. However, some of these values may not be physiologically relevant because *in vitro* systems can be exposed to concentrations higher than can occur *in vivo* in relevant tissues under conditions of a bioassay. Supplemental Material, Figure 1 (doi:10.1289/ehp.0901392) shows the number of hits per chemical as a function of the threshold AC_50_/LEC values used to define a hit. At the comparatively low threshold of 1 μM, there were relatively few hits per chemical. There were 828 chemical–assay pairs (0.5% of pairs tested) with an AC_50_/LEC < 1 μM (listed in Supplemental Table 2), many of which were related to nuclear-receptor–mediated xenobiotic metabolism. Of the chemicals that had AC_50_/LEC values < 1 μM in multiple assays, some showed cytotoxicity in one or more of the cell-based assays, which suggests cytotoxicity pathway activation, although in many cases we do not have a specific (cell-free) assay that would indicate which pathway that was. Cytotoxicity may comprise a relevant end point of specific biological process(es) leading to cellular demise (e.g., apoptosis), or it may comprise nonspecific collapse of cellular homeostasis (e.g., necrosis). Both are considered in phase I, and the former may be the result of targeted pathways engaged by specific molecular lesions, whereas the latter may generally follow from nonspecific cell injury. In other chemicals, we only saw specific targeted activities at these low concentrations, without any accompanying cytotoxicity.

Confidence in the predictive power of *in vitro* HTS data builds from many examples that confirm reported mechanisms of action for a number of well-studied chemicals. For example, bisphenol A, a known estrogen receptor (ER) agonist (Chapin et al. 2008), had AC_50_/LEC values < 1 μM for three separate ER (estrogen receptor, ESR1) assays [Supplemental Material, Table 2 (doi:10.1289/ehp.0901392)]. Expected ER activity at concentrations < 1 μM was also found for methoxychlor’s potent metabolite 2,2-bis(4-hydroxyphenyl)-1,1,1-trichloroethane. Similarly, results for the well-known androgen receptor (AR) antagonists linuron, prochloraz, and vinclozolin (Wilson et al. 2008) showed activity in AR assays (linuron, 57 μM antagonist, 5.1 μM binding; prochloraz, 12.5 μM binding; vinclozolin, 27 μM antagonist, 0.9 μM binding). Expected peroxisome proliferator–activated receptor (PPAR) activators perfluorooctanoic acid (PFOA) and perfluorooctane sulfonic acid (PFOS) (DeWitt et al. 2009; Lau et al. 2004), diethylhexyl phthalate (Melnick 2001), and lactofen (Butler et al. 1988) were all positive for PPARγ assays, and all but PFOS were also active in PPARα assays. Azoxystrobin, fluoxastrobin, and pyraclostrobin were active mitochondrial poisons in the HepG2 (hepatocellular carcinoma cell line G2) high-content cell-imaging assays, consistent with their pesticidal mode of action (Brandt et al. 1988). Thus, the redundancy and complementarity of multiple assays allow an integration of data across multiple assay technologies to boost confidence in the results. In some cases, *in vitro* results include indications of other biological pathways being activated by these well-studied chemicals, suggesting that other modes of action may be operative as well. To take one chemical as an example, PFOS shows activity against several matrix metalloproteinases, with AC_50_ values for direct interaction with matrix metalloproteinase (MMP)3 and MMP13 in cell-free HTS assays (14.6 and 32.4 μM, respectively) and perturbation of MMP1 and MMP9 levels in a cell-based assay (13.3 and 4.4 μM, respectively). MMPs are involved in the breakdown of extracellular matrix during development and tissue remodeling. These and other interactions could lead to the formation of specific hypotheses to test regarding toxicity mechanisms of these chemicals.

### Activity against human genes and pathways

Most of the ToxCast assays use human proteins and cells because our ultimate aim is to predict human toxicity. Assays probed 231 human genes either through direct interactions with the relevant protein or using a variety of indirect, downstream readouts of mRNA or protein levels. These genes were mapped to 143 published pathways from the KEGG (Kyoto Encyclopedia of Genes and Genomes) (Kanehisa et al. 2002) and Ingenuity Systems (http://www.ingenuity.com). From these human-based assays, composite gene and pathway perturbation scores were calculated. We computed “gene perturbation scores” for the subset of genes for which we had one or more assays, and these were assigned an LEC value for each chemical. The LEC is the minimum AC_50_/LEC value for that chemical in any assay that was mapped to that particular gene. We also computed “pathway perturbation scores,” which were assigned the minimum AC_50_/LEC value for a chemical in any assay that was mapped to a gene in the pathway. For a chemical to be considered active in a pathway, it had to have shown activity in at least five assays that mapped to that pathway. A total of 122 pathways had at least one chemical hit. [The chemical-by-pathway assay LEC values are given in Supplemental Material, Table 3 (doi:10.1289/ehp.0901392).] This collection of published pathways show significant overlap, so we also compiled a minimal set of 33 pathways inclusive of all genes represented in the total pathway set. Although this is a small subset of the total number of human pathways that could lead to toxicity, it allows us to sample the range of potential activities across phase I chemicals. Supplemental Figure 2 shows a network diagram of the minimal set of pathways linked to the genes for which we have assays. From this one can see redundancy between pathways in the down-selected target set.

**Figure 2:**
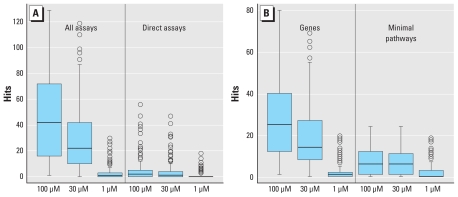
Distribution of number of hits per chemical as a function of AC_50_/LEC cutoff used to define a hit. (*A*) Distributions for all human assay measurements (out of 425) and the “direct” measurements from the cell-free HTS assays. The other assays are cell based and can potentially respond to multiple direct chemical interactions. (*B*) Number of hits per chemical for the gene and pathway perturbation scores. In each box and whisker plot, the heavy bar indicates the median, the boxes encompass the second and third quartiles, the whiskers extend to ±1.58 (interquartile range)/(number of assay-chemical hits), and the circles indicate outliers.

Figure 2 shows the distribution of hits across all assays, direct assays, and gene and minimal pathway perturbation scores, as a function of the minimum AC_50_/LEC value used to define a hit. Direct assays are those measuring perturbation of chemical–target activity in an optimized biochemical assay (Table 1). The balance of the assays are cell based and mostly measure up- or down-regulation of particular genes or proteins through direct or indirect mechanisms of chemical activity. Because indirect effects can arise from multiple direct chemical–target interactions, chemicals logically show broader activity in these assays. The number of direct assay and total assay measurements for human targets are 130 and 425, respectively. In general, the ratio of hits between direct and indirect is much less than the overall ratio of the number of direct to indirect assays. Some chemicals show a large number of hits against direct targets. At a 30-μM cutoff for activity, nine chemicals have at least 20 direct hits: emamectin benzoate, fentin, imazalil, mancozeb, maneb, metiram-zinc, milbemectin, oxytetracycline dihydrate, and PFOS. Mancozeb, maneb, and metiram-zinc are different salts of the same parent, and emamectin benzoate and milbemectin are related macrocyclic antibiotics. Overall, however, these nine chemicals are structurally diverse. Figure 2B shows the same distribution of hits for the gene and minimal pathway assays. Note that the scale for the pathways is significantly smaller because of the requirement that chemicals hit at least five pathway-mapped assays to be considered to have a positive pathway perturbation score. Except at the lowest cutoff of 1 μM, the median number of hits for genes or minimal pathways is > 5, and a number of chemicals show much broader activity than this. The chemicals that hit ≥ 20 of the minimal pathways with a 30-μM cutoff are fluazinam, mancozeb, maneb, metiram-zinc, and pyraclostrobin.

**Figure 3:**
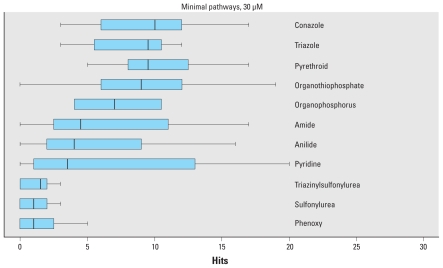
Distribution of number of hits against the 33 minimal pathways by chemical class (active at concentrations of < 30 μM). Only chemical classes with at least 10 chemicals are included. In each box and whisker plot, the heavy bar indicates the median, the boxes encompass the second and third quartiles, and the whiskers extend to ±1.58 (interquartile range)/(number of assay-chemical hits).

This broad range of activity is not seen universally across chemical classes. Figure 3 shows the distribution of hits against the minimal pathway set with chemicals parsed by chemical class (limited to classes with at least 10 chemicals). The conazoles and triazoles (many of which overlap) and pyrethroids show the broadest activity spectrum, with median number of pathway hits of around 10 of the 33 minimal pathways. In contrast, the sulfonylurea and phenoxy compounds are active in only a few pathways on average. However, even across the broadly active chemical classes, there is a spectrum of activity. These findings show that environmental chemicals are active across multiple human genes and pathways.

**Figure 4:**
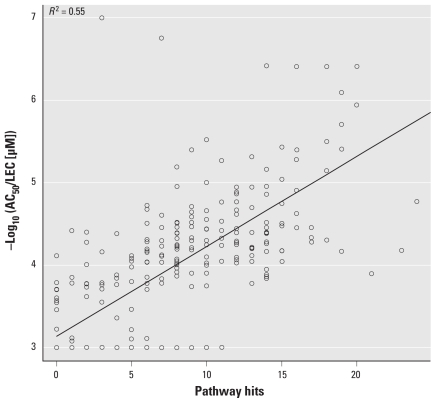
Plot of the minimum concentration at which a chemical caused cytotoxicity as a function of the number of minimal pathways in which the chemical was active at concentrations < 30 μM. Chemicals for which no cytotoxicity was observed were assigned an AC_50_ of 1 mM. The correlation coefficient is minimally sensitive to this default value. The line gives the fitted regression model.

We next examined the consequence of the multiplicity of pathways perturbed by these chemicals. A simple analysis is to see if the likelihood of cytotoxicity increases with the number of pathways in which a chemical is active. The data set includes 15 cytotoxicity assays using 11 primary human cell types or cell lines. We found a strong correlation between the number of pathways in which a chemical is called active and the minimum concentration at which cytotoxicity is observed across 15 cytotoxicity assays. Figure 4 shows the correlation between the number of pathway hits and the minimum AC_50_/LEC for cytotoxicity across the 15 assays. The *p*-value for the association is < 2.2E-16, and *R*^2^ = 0.55 for linear correlation.

We tested the hypothesis that the lower the concentrations at which a chemical shows activity *in vitro*, the lower will be the doses at which *in vivo* toxicity will be observed for that chemical. This hypothesis is based on three assumptions: *a*) Pathways perturbed by a chemical *in vitro* will also tend to be perturbed *in vivo*, although the magnitude may be very different because of tissue-specific feedback or adaptation not active *in vitro. b*) Pathway perturbations *in vivo* arising from specific chemical–target interactions require chemical concentration at the target site to be in the range where effects on the *in vitro* assay are seen; hence, lower *in vitro* AC_50_ values imply lower concentrations at which *in vivo* effects are seen. *c*) There are combinatorial pathways that, when perturbed, can lead to a given observed toxicity, and the AC_50_ values for the toxicity-related pathways for a chemical will be distributed randomly through the total distribution of AC_50_ values, including some in the low concentration tail of that distribution.

To test this hypothesis, we first looked for direct correlations between low *in vitro* pathway perturbation score AC_50_ values for the minimal pathway set and the lowest dose at which toxicity was seen *in vivo*. Because we have only sparsely sampled the space of direct targets (e.g., enzymes, receptors), we used the number of pathways perturbed below some concentration threshold as a surrogate estimate for minimum concentration at which a chemical significantly perturbs pathways. This is based on the assumption that each chemical shows a distribution of AC_50_ values across the complete set of pathways and that this distribution has a long tail going toward low concentrations. More pathway hits below a defined cutoff will correlate with the entire distribution shifting toward lower concentrations. For each chemical and each *in vivo* study type in ToxRefDB, we tabulated the lowest dose at which any treatment-related effect occurred. A linear regression fit between the number of pathway hits at concentrations < 30 μM (trend and significance is relatively insensitive to this cutoff) and the lowest dose at which toxicity was observed yielded *p*-values of 0.0031 (chronic rat), 0.0007 (chronic mouse), 0.037 (developmental rat), 0.053 (developmental rabbit), and 0.019 (multigenerational rat). Except for the developmental rabbit study, all study types showed a significant association at the 0.05 level. In addition, the sign of the association was correct in all cases: The higher the number of low-concentration *in vitro* pathway hits, the lower the observed lowest toxic dose *in vivo*. Therefore, these results show a significant association between low *in vitro* concentrations for pathway perturbations caused by a chemical and the lowest dose at which treatment-related effects are first seen *in vivo*.

**Figure 5:**
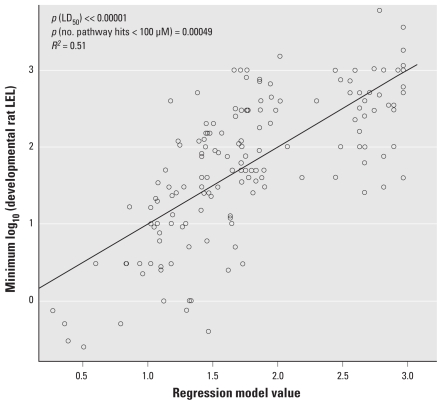
Association between the number of minimal pathway hits (which we assume is inversely correlated with the minimum concentration at which significant pathway activity occurs for the chemical) and the lowest dose *in vivo* at which a significant toxicity end point is observed, in this case for the rat prenatal developmental bioassay. Each point represents a single chemical. The *x*-axis is the value resulting from the fitted model, which is 0.6 + 0.4 × log_10_(LD_50_) – 0.037 × (number of minimal pathway hits at concentrations < 30 μM). The *y*-axis is the minimum log_10_(concentration) at which toxicity is seen for this study type. This analysis was performed on the 153 chemicals for which we had all values.

We also performed the association calculation using the short-term half-maximal lethal dose (LD_50_) (International Programme on Chemical Safety 2005) as a covariate. LD_50_ has a strong correlation with the lowest dose at which other toxic effect occurs and can help correct for factors not included in the pathway parameter, including pharmacokinetics. In models including both terms, the *p*-values for association between the number of pathway hits at concentrations < 30 μM and the lowest dose at which toxicity was observed were 0.0019 (chronic rat), 0.00015 (chronic mouse), 0.00049 (developmental rat), 0.011 (developmental rabbit), and 0.00063 (multigenerational rat). We see stronger correlations between *in vitro* activity and the threshold of toxicity after adjusting for LD_50_, and the sign of the effect was as hypothesized in all cases. The example in Figure 5 shows the results of the model fit for prenatal developmental toxicity in rats, which resulted in the highest correlation across the five study types (*R*^2^ = 0.51).

### Rat liver tumors and PPAR signaling

Almost half of the tested chemicals caused tumors in either rats or mice in high-dose 2-year chronic/cancer bioassays (Martin et al. 2009a), with most of these having been determined by the U.S. EPA to be nongenotoxic tumorigens (U.S. EPA 2009). Of the 309 chemicals tested, 248 have rat 2-year chronic/cancer bioassay data entered into ToxRefDB, and 21 of these are liver tumorigens [chemicals shown in Supplemental Material, Figure 3 (doi:10.1289/ehp.0901392)]. These 21 are a subset of the 97 chemicals that are rat tumorigens of any tissue type. All rat liver tumors caused by this set of chemicals were hepatocyte derived. We tested for univariate associations of all *in vitro* assays and gene perturbation scores against all rodent liver *in vivo* end points, and identified a total of five *in vitro* assays with a significant association with rat liver tumors (Fisher’s exact test *p*-value < 0.01). Results for these five assays and for the 21 chemicals that are rat liver tumorigens are illustrated in Supplemental Material, Figure 3. Three of the five assays are associated with the nuclear receptor pathway genes *PPARA* and *PPARG*, one is associated with the cytokine chemokine (C-C motif) ligand 2 (*CCL2*), and the last with the AR. The *PPARA* transcription reporter assay shows high specificity (0.99) but low sensitivity (0.19) (Fisher’s exact *p*-value = 0.0005). The relative risk of causing rat liver tumors for chemicals being positive for this assay was 9.5. The *PPARG* assay shows high sensitivity (0.86) but low specificity (0.53) (Fisher’s exact *p*-value = 0.0009). Also associated with rat liver proliferative lesions is hydroxymethylglutaryl-coenzyme A synthase 2 (*HMGCS2*), which is a gene regulated by *PPARA*, providing indirect evidence that the human PPARα pathway has been activated by this group of chemicals.

PPAR activation is a well-studied mechanism or mode of action for chemically induced liver tumors in rodents (Abbott 2008; Klaunig et al. 2003; Lai 2004; Peters 2008; Takeuchi et al. 2006). The primary role of PPARs is in lipid and fatty acid metabolism; however, xenobiotic compounds may activate PPAR in hepatocytes, leading to induction of xenobiotic metabolizing enzymes as well as peroxisome proliferation and hepatocyte hypertrophy. During prolonged exposure to PPAR activators, rodent hepatocytes can become hyperplastic, necrotic, or apoptotic, and in some cases neoplastic. The relevance of PPAR-mediated rodent tumors to human toxicity and disease is an active area of research and debate (Desvergne et al. 2009; Guyton et al. 2009; Klaunig et al. 2003). Nonetheless, based on the carcinogenic potential of PPAR-activating compounds, current U.S. Food and Drug Administration (FDA) guidance on PPAR agonists requires 2-year carcinogenicity evaluations in rats and mice before initiation of human clinical studies longer than 6 months (U.S. FDA 2008).

CCL2 levels have been shown to be associated with severity or progression in a number of tumor types (Roca et al. 2008). CCL2 helps drive angiogenesis (Kuroda et al. 2005). There is also evidence linking CCL2 with up-regulation of bile acids, cholestatic liver injury, and fibrogenesis in rats (Ramm et al. 2009). Studies have discovered linkages between AR and androgen levels and hepatocellular carcinoma in humans and animals [reviewed by Kalra et al. (2008)].

There is extensive evidence that perturbing androgen signaling activity is associated with increased risk of liver tumors. AR is expressed in the liver of rats (Konoplya and Popoff 1992) and humans (Iqbal et al. 1983), and hepatic tumor development is likely influenced by androgens, as indicated by the fact that males have a greater prevalence of liver neoplasms in humans (Curado et al. 2007) and rodents (Kemp and Drinkwater 1989). Elevated levels of testosterone (Grange et al. 1987) are associated with increased risk of hepatic adenomas in men. In male rats, testosterone (Morris and Firminger 1956) promote rat liver tumor development. The hypothesized mode of action for the liver tumorigenicity of AR antagonists such as vinclozolin and linuron is as follows: The antiandrogens block AR function and negative feedback of the pituitary, so more luteinizing hormone is produced, which in turn leads to increased production of androgens by testicular Leydig cells. Whereas androgen homoeostasis may eventually reset, animals can have significantly elevated androgen levels, which can in turn promote liver tumor development.

**Figure 6:**
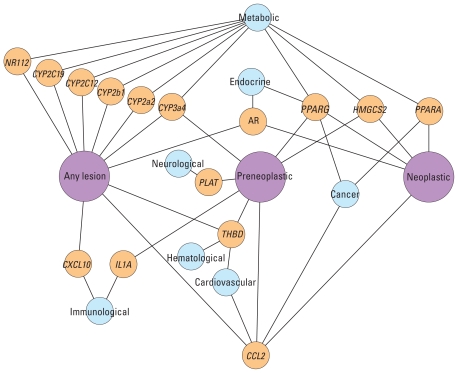
Network of genes associated with the progression of rat liver tumor end points. Associations were calculated using Fisher’s exact test, with assay AC_50_/LEC values ≤ 100 μM set to 1 and those with > 100 μM set to 0. Only associations with a *p*-value < 0.01 are included. Links between genes (yellow) and *in vivo* end points (pink) are shown where there is a statistical association based on the *in vitro* assay results. The “Any lesion” category contains the “Preneoplastic” category, which in turn contains the “Neoplastic” lesions category. Disease or disorder classes (cyan) are linked to genes according to Goh et al. (2007).

We also investigated associations between *in vitro* assays and the progression of liver disease in rats. Chemicals were categorized according to rat liver disease progression: those causing no liver lesions (122 chemicals) or causing any type of liver lesion (126 chemicals). Chemicals causing liver lesions could be classified further into subsets of those causing preneoplastic or neoplastic liver lesions (58 chemicals), or those causing just neoplastic liver lesions (21 chemicals). All assays were correlated against these three rat liver lesion categories. Figure 6 shows associations with a *p*-value < 0.01 (either *t*-test or Fisher’s exact test), in which the genes linked to assays statistically associated with the three rat liver lesion categories, as well as human disease categories assigned through the Online Mendelian Inheritance in Man (OMIM) database (Goh et al. 2007). *PPARG*, *HMGCS2*, and *CCL2* are all associated with preneoplastic and neoplastic levels in the liver disease progression, and *PPARA* is additionally associated with neoplastic lesions.

More than half of genes with any association were involved with xenobiotic metabolism in the liver (9 of 15), with most of these being cytochrome P450 enzymes. Many of these XME genes are regulated by PPAR or other nuclear receptors, and other assays indicated direct associations with rat and human pregnane X receptor (NR1I2). Preneoplastic and neoplastic liver lesions are also associated with *PPARG* activation. These data suggest that induction of liver neoplasms by these chemicals is *PPARA* dependent, and potentially coupled with PPARG and CCL2, whereas a variety of xenobiotic metabolism and other pathways can lead to more general liver lesions.

## Discussion

The large ToxCast data set links *in vitro* and *in vivo* assay results to genes and pathways, providing a unique public resource for researchers modeling chemical biology and toxicity. We are expanding this collection in both chemical and assay space and plan to test thousands of environmental chemicals in the coming years. The examples we give here are among the many areas of toxicology that can be explored using this data set, and we are finding other associations with chronic, developmental, and reproductive toxicity. *In vitro* assays directly probe chemical perturbations of pathways either by measuring small molecule–protein interactions or closely linked downstream effects. Because of this, we can make use of information on links between genes, proteins, and diseases that have been derived from genetic variation and gene knockout studies. Organizing HTS *in vitro* data around human toxicity and disease pathways will allow synthesis with other mechanistic data on environmental chemicals coming from genomics, proteomics, and metabolomics studies. An initial mapping of this set of assays to broad molecular, cellular, and disease classes using the OMIM-based categories of Goh et al. (2007) is illustrated in Supplemental Material, Figure 4 and Table 4 (doi:10.1289/ehp.0901392). Genes in the current assay set are linked to various toxicity end-point classes. One important series of next steps is to identify the key disease classes and pathways relevant to the toxicity of environmental chemicals and to work with other researchers to develop critical missing assays in these pathways.

Our short-term goal is to screen large numbers of environmental chemicals and prioritize them for further testing, based on scores for disease-related predictive signatures and on exposure potential. The longer term goal is to use *in vitro* assays to understand the multitude of mechanisms of action for *in vivo* chemical toxicity, and for this to be realized there remain a number of significant challenges. The most widely held criticism of this *in vitro*–to–*in vivo* prediction approach is that genes or cells are not organisms and that the emergent properties of tissues and organisms are key determinants of whether a particular chemical will be toxic. A related challenge is the understanding of what short-timescale (hours to days) *in vitro* assays can tell us about long-timescale (months to years) processes that lead to *in vivo* toxicity end points such as cancer. Finally, biotransformation of compounds into metabolites that can be more or less active than the parent clearly must be considered in the assay or modeling treatment. We either need assays in which realistic levels of biotransformation occur *in vitro* so that the complete suite of active metabolites can be assessed, or need to explicitly or implicitly test active metabolites.

Understanding the correlation between *in vitro* AC_50_/LEC values and the corresponding chemical concentrations in blood or tissues will be crucial in extending this approach to quantitative risk assessments. If we find that environmental contaminants activate toxicity pathways in cell systems at concentrations close to those detected in human samples, for instance, from population-wide biomonitoring studies (Centers for Disease Control and Prevention 2005), it should raise the priority for studying the potential human toxicity of those chemicals (National Research Council 2007). In ToxCast, we aim to predict the potential for chemicals to affect human health, but all of the current *in vivo* data being used to develop prediction models is from high-dose animal testing. Where possible, it will be important to evaluate chemicals for which we have human toxicity data, such as pharmaceutical compounds that have displayed toxicities when tested in humans. The U.S. EPA and Pfizer Inc. have recently agreed to work together in testing such compounds in the next phases of ToxCast. Assuming these challenges are adequately addressed, we believe that this HTS approach for toxicity testing will be a practical solution for evaluating the backlog of thousands of untested environmental chemicals, leading to more efficient, informed, and targeted testing for protection of public health.

## Conclusions

The first phase of ToxCast, outlined here, is an important step in evaluating the use of high-throughput *in vitro* assays to prioritize chemicals for more detailed testing and to prioritize which tests should be run. The latter will be driven by the mechanistic understanding that these assays provide. Perhaps the most important conclusion from the summary data presented here is how multifunctional these chemicals can be. Chemicals can hit many molecular targets and perturb many pathways, albeit typically with AC_50_ values of tens of micromolar. This means that understanding the route from molecular interactions to *in vivo* toxicity will likely not be a matter of finding single molecular targets linked to well-defined whole-animal phenotypes. Whether at the molecular, cellular, tissue, or whole-animal level, these chemicals have the potential to perturb many processes.

Understanding the complex biological cascades triggered by environmental chemicals and understanding how to use *in vitro* data in a prioritization and regulatory context will be complex tasks requiring insights spanning many disciplines. Because of the enormity of the challenge, we have already made the ToxCast phase I assay data available to a network of analysis partners around the world. These results are being compared with the $2 billion worth of traditional toxicology results, collected by the U.S. EPA over the past 30 years and incorporated into ToxRefDB, as a transitional step toward a new toxicity testing paradigm focused on predicting the potential hazards of environmental chemicals. When key events are linked to toxicity and disease pathways, they provide regulatory agencies with a powerful new tool for determining under what conditions environmental exposures pose risks to human health.

The ability to use molecular and computational sciences holds the potential to usher in a new era of prioritizing, assessing, and managing chemicals at the U.S. EPA. Building this new toxicity testing paradigm will be a challenge and will take time, and no one organization can accomplish it alone. In addition, achieving these objectives will require transparency, data sharing, peer review, and a cohesive plan for interpretation and application of these emerging approaches. We are preparing to launch a second phase of ToxCast that will expand on and verify the ability of this approach to predict potential human toxicity. We expect to complete this second phase of ToxCast over the next several years and realize the promise of delivering innovative computational methods for evaluating potential health impacts of environmental chemicals.

## Figures and Tables

**Table 1:** Summary of the ToxCast *in vitro* assays: types of cells, number of concentrations (concentration range), time points, and types of readout

Assay set	Assays	Cell type	Concentrations (μM)	Time points	Readout
Cell-free HTS	239	Cell free	CYP assays: 8 (0.00914–20) All others: 8 (0.0229–50)	1	IC_50_
Cell-based HTS	13	HEK293, HeLa, HepG2, FAO	15 (0.0012–92)	1	IC_50_
High-content cell imaging	19	HepG2 and primary rat hepatocytes	10 (0.39–200)	3 (1, 24, 72 hr)	IC_50_
Quantitative Nuclease protection	16	Primary human hepatocytes	5 (0.004–40)	3 (6, 24, 48 hr)	IC_50_
Multiplex transcription reporter	81	HepG2	7 (0.0014–100)	1	LEC
Biologically multiplexed activity profiling (BioMAP)	87	HUVEC, HDFn, HBEC, ASMC, KC, PBMC	4 (1.48–40)	1	LEC (separate up- and down-regulation readouts)
Phase I and II XME cytotoxicity	4	Hep3B	9 (0.0146–960)	1	IC_50_
HTS genotoxicity	1	TK6	3 (50–200)	1	LEC
Real-time cell electronic sensing	7	A549	8 (0.047–100)	Continuous (0–48 hr)	IC_50_, LEC

Abbreviations: A549, human alveolar basal epithelial cell carcinoma cell line 549; ASMC, arterial smooth muscle cells; CYP, cytochrome P450; FAO, Reuber rat hepatoma cell line; HBEC, human bronchial epithelial cells; HDFn, human neonatal foreskin fibroblasts; HEK293, Human embryonic kidney cell line 293; HeLa, Henrietta Lacks cervical cancer cell line; Hep3B, hepatocellular carcinoma cell line 3b; HepG2, hepatocellular carcinoma cell line G2; HUVEC, human umbilical vein endothelial cells; KC, keratinocytes; PBMC, peripheral blood mononuclear cells; TK6, T-cell blast cell line 6. Data were collected in concentration–response format for each chemical–assay pair. If data were fit to a Hill function, we report the AC_50_ values. In other cases, an LEC was determined by significant change relative to negative control. Assay methods are described in more detail in Supplemental Material (doi:10.1289/ehp.0901392).
